# Supplementary material for: Environmental effects on explosive detection threshold of domestic dogs
Source: PLoS One. 2024 Sep 25;19(9):e0306817. doi: 10.1371/journal.pone.0306817 (PMC11423966; doi:10.1371/journal.pone.0306817)
Supplement: S1 Table — Scoring for this scale ranges from 0 to 10. Zero being defined as resting and breathing is an involuntary process, and ten being very heavy breathing and the chest and abdomen are expanding and collapsing violently. (DOCX) [file pone.0306817.s001.docx]

Supplemental Table 1

**Table 1: Description of RE scores**

| PRE Score | Definition |
| --- | --- |
| 0 | Dog is in a calm state and resting. Respiration is involuntary and chest expands lightly. |
| 1 | Dog is in a calm state and resting. The chest expands slightly more noticeably, and respiration is a voluntary process. |
| 2 | The dog is not resting but is in a calm state. The chest noticeably expands and collapses to deliver more air to the lungs. |
| 3 | Dog is slightly aroused. There is no panting, but the chest expands and collapses with greater momentum. The abdomen is not engaged in the respiration process. |
| 4 | Dog is in an aroused state and is panting, however tongue remains retracted inside the mouth. The chest expands and collapses with moderate effort. The abdomen is engaged in respiration. |
| 5 | Dog is in an aroused state and panting. The tongue is extended out of the mouth. The chest and expands and collapses with moderate effort. Abdomen is engaged in respiration process. |
| 6 | Dog is panting heavily. The tongue is extended outside of the mouth and the base of the tongue is more bulbous. The chest expands and collapses with great effort and abdomen is engaged in respiration. |
| 7 | Dog is panting heavily. The tongue is extended outside of mouth and the base is bulbous. Liquid is gathering and dripping from the tip of the tongue. The chest expands and collapses with maximal effort. The abdomen is engaged and contracting intensely. |
| 8 | Dog is panting heavily, and tongue is fully extended outside of the mouth. The tongue falls to the side of the mouth and is extremely bulbous. Liquid is dripping from the tongue and excess foam is forming. The chest expands and collapses with maximal effort and abdomen is contracting intensely. |
| 9 | Dog is panting heavily and salivating excessively. Tongue is fully extended outside of the mouth and falls to the side of the mouth. The tongue is extremely bulbous, and liquid is dripping from the tongue with excess foam is forming. Both the chest and abdomen expand and collapses violently. |
| 10 | Dog is breathing with maximum effort. The dog is forcibly panting heavily and salivating excessively. The tongue is fully extended outside of the mouth and falls to the side of the mouth. The tongue is extremely bulbous, and liquid is dripping from the tongue with excess foam is forming. Both the chest and abdomen expand and collapse violently. |
